# Medication-related osteonecrosis of the jaw in cancer patients: Case series and review of the current literature

**DOI:** 10.21142/2523-2754-1003-2022-123

**Published:** 2022-09-28

**Authors:** Karen Maciel Reyes Castillo, Miguel Ángel Ocampo Benítez, Omar Peña Curiel

**Affiliations:** 1 Universidad Nacional Autónoma de México. Coyoacán, México. kren_93_14@hotmail.com Universidad Nacional Autónoma de México Universidad Nacional Autónoma de México Coyoacán Mexico kren_93_14@hotmail.com; 2 Centro Médico Nacional 20 de Noviembre del ISSSTE. Ciudad de México. maxilocampo@outlook.com Centro Médico Nacional 20 de Noviembre del ISSSTE Ciudad de México maxilocampo@outlook.com; 3 Centro de Cáncer de Mama TecSalud. Nuevo León, México. omar.oncologia@outlook.com Centro de Cáncer de Mama TecSalud Nuevo León México omar.oncologia@outlook.com

**Keywords:** medication-related osteonecrosis, bone-modifying agents, zoledronic acid, denosumab, bevacizumab, osteonecrosis relacionada con medicamentos, agentes modificadores óseos, ácido zoledrónico, denosumab, bevacizumab

## Abstract

Currently, a large proportion of cancer patients are treated with bone modifying agents (BMA). In this regard, the increase in the prescription of these drugs has lead to concerns in the increment of osteonecrosis of the jaws. This article describes four patients with BMA cancer treatments requested dental evaluation at our institution due to pain and swelling of the mandibular bone after tooth extraction, tooth loss, or unknown risk factor. Oral and radiographic evaluation reveals Medication-related osteonecrosis of the jaw (MRONJ) at different clinical stages according to the American Association of Oral and Maxillofacial Surgeons (AAOMS) classification. Some patients underwent abscess drainage, oral cleaning and antibiotic therapy with complete recovery. Follow-up showed treatment success in all patients. That is why we emphasize the importance of early establishment of appropriate treatment and emphasize the avoidance of dental procedures during BMA therapy.

## RESUMEN

En la actualidad, una gran cantidad de pacientes con cáncer son tratados con agentes modificadores óseos (AMO). En relación con esto, el aumento en la prescripción de estos fármacos ha generado preocupación por el incremento en la osteonecrosis de los maxilares. Este artículo describe cuatro casos de pacientes con cáncer, tratados con AMO, que acuden a nuestra institución debido a que padecen dolor e inflamación de la mandíbula, después de la extracción dental, así como pérdida de dientes con un factor de riesgo desconocido. La evaluación clínica y radiográfica evidenció osteonecrosis de los maxilares en diferentes etapas clínicas, según la clasificación de la Asociación Estadounidense de Cirujanos Orales y Maxilofaciales (AAOMS). Algunos de los pacientes fueron sometidos a drenaje de abscesos, limpieza bucal y antibioticoterapia con recuperación completa. El seguimiento clínico fue exitoso en todos los pacientes. Es por eso que hacemos énfasis en la importancia de establecer un tratamiento apropiado y evitar procedimientos dentales durante la terapia con AMO.

## INTRODUCTION

Cancer is one of the major health problems in the world. In 2020, more than 19 million new cases and almost 10 million deaths were reported globally ^1^. Cancer survivorship has shown a continuous increment in the past decade ^2^. This increase in patient survival has made the clinicians aware of the prevalence of late and chronic cancer treatment complications. These complications usually appear secondary to the development of distant organ metastases or as a consequence of the drug-related toxicity caused by chronic pharmacologic exposure.

With increasing survival of cancer patients, a large proportion of this population will eventually develop metastatic bone disease and will often suffer skeletal-related events (SREs) such as pain, hypercalcemia, or pathologic fractures ^3^. For the treatment of metastatic bone disease, bone-modifying agents (BMA) are usually indicated such as denosumab, a monoclonal antibody directed against RANK ligand (RANKL), or bisphosphonates (e. g. zoledronic acid, alendronate), a group of drugs that inhibit the maturation and recruitment of osteoclasts via suppression of the mevalonate pathway. Both drugs have been shown to be useful in decreasing SREs due to metastatic bone disease ^4^. Likewise, in some patients with advanced cancer, the combination of bevacizumab, a monoclonal antibody directed against vascular-endothelial growth factor, with chemotherapy has been shown to be useful for increasing progression-free survival, in some cancer types ^5^. In this regard, the augmented prescription of these drugs has led to concerns in the increment in drug-related toxicity such as medication-related osteonecrosis of the jaw (MRONJ).

According to the American Association of Oral and Maxillofacial Surgeons (AAOMS), a case of MRONJ is defined as an exposed bone in the maxillofacial region that has persisted for more than 8 weeks in a patient with previous or current treatment with a BMA or an antiangiogenic agent, without a history of radiotherapy to the head-and-neck region or mandibular bone metastatic disease. In addition, the AAOMS has established management strategies according to the MRONJ stage ranging from conservative treatment in early stages to surgical debridement and reconstruction for advanced stages ^6^.

In this article we present, a case-series of four patients with MRONJ in different clinical stages according to the AAOMS classification. Likewise, we review the clinical risk factors associated with each event and delineate the diagnostic and therapeutic approach to the patient with suspected MRONJ. The present article aspires to give practical recommendations for the general practice dentist.

## CASES DESCRIPTION

The patients gave their authorization for the reporting of some of their records and the work complied with the ethical standards of Helsinki.

Case 1 - A woman with lung cancer treated with antiangiogenic and antiresorptive agents.

A 70-year-old woman diagnosed with advanced non-small cell lung cancer with multiple bone, lung and tumor left adrenal gland metastases. The patient received cytotoxic treatment with carboplatin, pemetrexed and bevacizumab for one year, followed by denosumab for the last three months of follow-up. After the third administration of denosumab, the patient reported dental extraction of the left mandibular second molar and right mandibular first molar was performed outside of our institution. One month later, the patient began with severe pain in the manipulated area, delayed dental healing and paresthesia on the right-side of the mandible.

**Figure 1:**
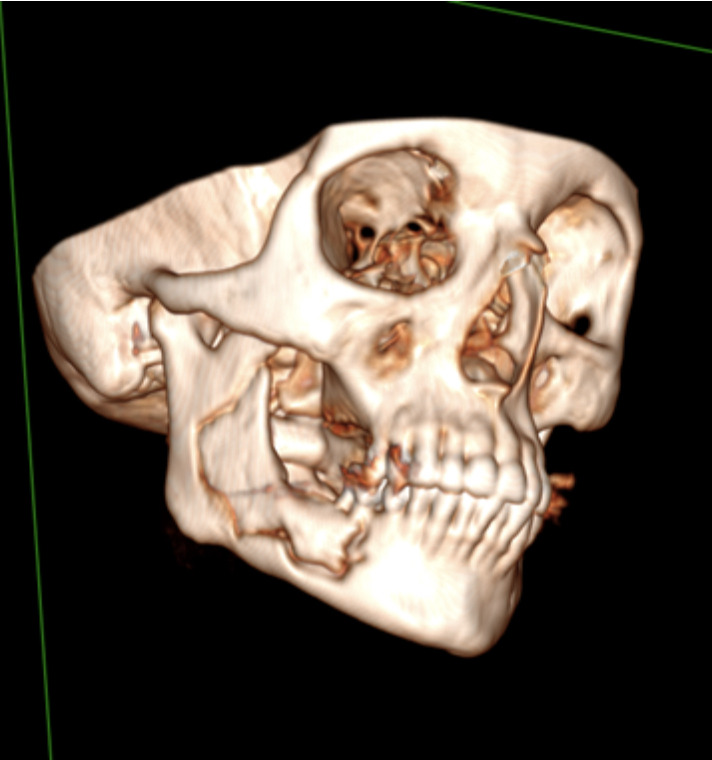
3-D computed tomography reconstruction shows an osteolytic lesion extending from the mandibular body to the right-side ascending ramus.

Intraoral examination showed exposure of necrotic bone and purulent secretion with inflammation of the surrounding molar area. Imaging studies showed an osteolytic lesion with changes in the bone trabecular pattern, extending from the mandibular body to the right ascending ramus (Figure 1). Based on these clinical characteristics, we classified the lesion as an AAOMS stage 3.

Treatment for three months with clindamycin was indicated together with mouth rinses with 0.12% chlorhexidine antiseptic solution. The infectious process, pain and the necrosis extension were successfully resolved.

Case 2 - A man with multiple myeloma treated with zoledronic acid and prednisone. 

A 56-year-old man diagnosed with multiple myeloma associated with bone metastases. Hematologic treatment consisted on the administration of thalidomide, cyclophosphamide, prednisone, and zoledronic acid for ten months.

**Figure 2:**
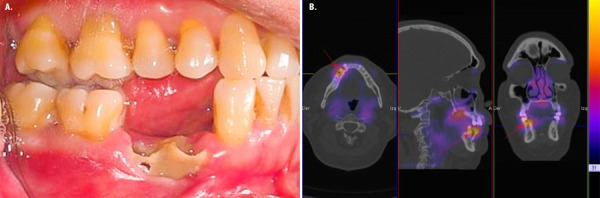
A. Exposure of necrotic alveolar bone after dental extraction on premolar area. B. Spect CT shows defined extension of osteonecrosis in right mandible and demonstrated hypermetabolism evident in right-side (Red arrow).

A dental extraction of the right mandibular first premolar was performed outside of our institution. A week later, the patient referred spontaneous loss of the second mandibular right premolar in addition to paresthesia in the inferior border of right mandibular body. Intraoral examination showed exposure of necrotic alveolar bone in the affected area (Figure 2A ).

The orthopantomography study showed absence of bone remodeling. Furthermore, a SPECT-CT was performed where hypermetabolism was evident in the affected mandibular body (Figure 2B). The lesion was consistent to an AAOMS stage 2 MRONJ and received amoxicillin / clavulanate for three months with full recovery.

Case 3 - A man with multiple myeloma treated with zoledronic acid and corticosteroids. 

A 74-year-old man diagnosed with multiple myeloma and multiple bone metastases was treated with thalidomide, dexamethasone, cyclophosphamide, and zoledronic acid for 19 months.

**Figure 3:**
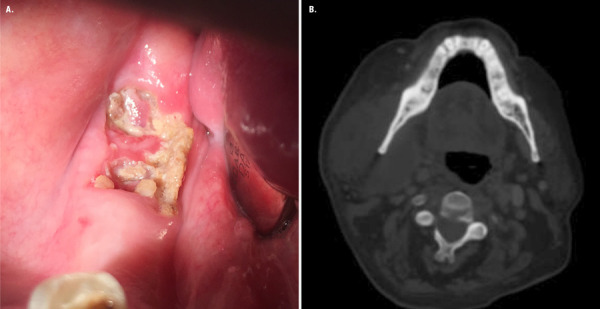
A. Intraoral examination showing necrotic bone exposure in right-side third molar region. B. Axial CT image shows the extension of submandibular space infection on the right-side.

The patient reported abrupt ongoing pain in the mandibular area and trismus. Intraoral examination showed necrotic bone exposure and purulent exudate in the right molar area and swelling in the submandibular right side on extraoral examination (Figure 3A).

Orthopantomography and head and neck CT scan were performed on suspicion of a submandibular abscess. An osteolytic lesion was observed in the right molar area and a submandibular abscess (Figure 3B). Based on the clinical characteristics, the patient was classified as AAOMS stage 3.

The patient underwent abscess drainage followed by broad-spectrum antibiotic therapy with vancomycin and ertapenem for one week. After a microbial cultures Streptococcus intermedius was isolated and antimicrobial therapy was adjusted with cephalexin for one week and mouthwashes with 0.12% chlorhexidine solution for 15 days. Pain relief and full recovery was obtained.

Case 4 - A woman with breast cancer treated with long-term zoledronic acid. 

A 70-year-old woman with advanced breast cancer and multiple bone metastases was treated intermittently with zoledronic acid for four years. With a sudden onset, the patient reported pain in the right mandibular area after spontaneous loss of canine tooth. Intraoral examination showed a submental and alveolar ridge fistula with purulent exudate.

**Figure 4:**
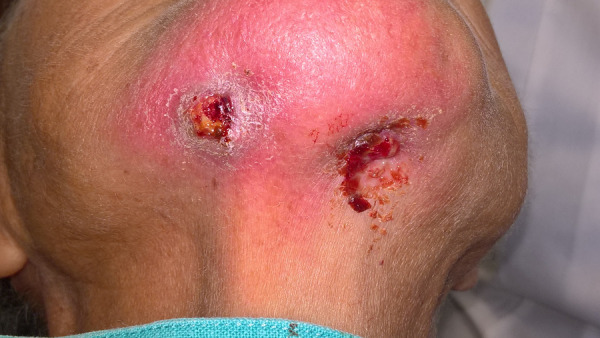
Extraoral examination demonstrated the cutaneous sinus tract in the submental region.

Antimicrobial treatment was initiated with clindamycin and mouthwashes with 0.12% chlorhexidine solution. Four months later, examination showed an second extraoral fistula on the right submental area with purulent discharge upon palpation. (Figure 4).

Orthopantomography showed an osteolytic lesion in the lower border of the mandibular body. The patient started treatment with clindamycin for 2 weeks, mouthwashes with 0.12% chlorhexidine solution three times daily for 15 days.

After a month of treatment, she showed improvement of symptoms and the cutaneous fistula had healed without complications.

## DISCUSSION

In this case series we present four patients with MRONJ associated with denosumab, bisphosphonates, and bevacizumab exposure, three of the drugs most commonly associated with this complication and also three of the drugs most commonly used in daily oncologic practice in the midst of advanced disease in different tumor types.

The reason underlying the almost exclusive maxillary bone affection is still unknown. In preclinical models, it has been observed that the maxillary bones show an increased bone-remodeling activity owing to the frequent microtrauma inherent to that specific area, thus making the maxillary bones more susceptible to the inhibition of bone remodeling by antiresorptive and antiangiogenic agents. Moreover, Cheong et al. observed a direct soft tissue toxic effect with bisphosphonate exposure in a murine model favoring bacterial colonization and infections in the gingival surface ^7^. Additionally, VEGF is essential for the bone remodeling process as it has been shown to promote osteoclast differentiation and survival ^8^. Therefore, inhibition of VEGF could delay repair during wound healing and bone remodeling and has been shown to play an important role in the development of MRONJ ^9^.

The incidence of MRONJ depends on the type of medication administered (antiresorptive vs. antiangiogenic agents), the indication (osteopenia/osteoporosis vs. bone metastases), and the length of exposure to the drug ^6^. In this manner, bisphosphonate-associated MRONJ leads the incidence of this complication with 0.7 to 6.7% of exposed patients, followed by denosumab 0.7 to 1.9%. Contrariwise, the percentage of anti-angiogenic-associated MRONJ is only 0.2% of exposed patients.^10^^-^^12^ This calls for special attention to clinical symptoms in bisphosphonate-exposed patients.

It is well known that MRONJ occurs almost exclusively in patients with previous dental alterations, either inflammation (e. g. periodontitis) or secondary to dental extractions performed during concurrent treatment with any of the drugs associated with MRONJ. Furthermore, the risk of developing MRONJ after a dental extraction and concurrent exposure to an BMA is estimated to be around 2.8%, although it has been reported to be as high as 14.8% ^13^^,^^14^. Likewise, several other risk factors for the development of MRONJ have been identified. In a case-control study of 191 cases of MRONJ, Barasch et al. observed that the three most important risk factors associated with osteonecrosis were: 1) oral suppuration (periodontal disease), OR 7.8 [95% CI: 1.8-34.1; 2) dental extractions, OR 7.6 [95% CI: 2.4- 24.7], and 3) exposure to therapeutic radiotherapy to the mandible as part of head and neck cancer treatment, OR 24.1 [95% CI: 4.9-118.4] ^15^. Additionally, other authors have observed an increase in the risk of MRONJ in patients with chronic corticosteroid treatment, advanced age, and concurrent conditions such as diabetes mellitus, and tobacco consumption ^16^.

The AAOMS has developed clinical practice guidelines for the prevention, diagnosis, and treatment of MRONJ. With regard to prevention, several authors ^17^^-^^19^ recommend a thorough dental evaluation before initiating any drug associated with the development of MRONJ (Table 1). Moreover, studies by Dimopoulos, Bonacina, and Vandone et al. found a reduction of up to 50% of the incidence of MRONJ in patients exposed to antiresorptive agents when general preventive measures were applied prior to initiation, and throughout treatment. These measures included a thorough review by a certified dentist, education of the patient in oral hygiene, and the completion of surgical procedures 14 to 21 days before starting the treatment with antiresorptive agents ^20^^-^^22^.

**Table 1:** MRONJ preventive measures according to AAOMS

1.	Thorough examination of the oral cavity.
2.	Radiologic evaluation (as needed).
3.	Identify acute infection sites and potential infections sites and treat accordingly.
4.	Education and motivation of the patient regarding optimal oral hygiene.

Adapted from: Ruggiero et al.^6^ J Oral Maxillofac Surg 2014; 72:1938-1956

Despite the well-known preventive measures, a small percentage of patients still develop MRONJ. For these patients, urgent dental intervention is warranted in order to limit the extension of the lesion and, in selected cases, perform surgical procedures. The AAOMS treatment guidelines for MRONJ emphasize on: 1) eliminating pain, 2) controlling soft tissue infection, and 3) minimizing the progression and extent of osteonecrosis. The single most important therapeutic measure is the initiation of broad-spectrum antibiotics and thorough cleansing of the affected area with antiseptic solution followed by surgical debridement in selected cases depending on the extent of the osteonecrotic lesion ^19^. Recently, non-invasive therapies have been reported to limit the spread of MRONJ. Pentoxifylline and tocopherol can be useful in non-invasive management. Furthermore, low-level laser therapy (LLLT) and teriparatide, have also gained attention as potential non-invasive therapies for MRONJ. Furthermore, once MRONJ is resolved, strengthening of oral hygiene is of utmost importance ^23^^,^^24^.

## CONCLUSIONS

MRONJ is a relatively common complication associated with the prescription of bone-modifying and antiangiogenic agents in cancer patients. In this regard, it is important to be informed concerning the prevention and treatment of this complication. It is also important to be aware of the commonly MRONJ-associated pharmacologic agents and to avoid surgical oral manipulation during concomitant treatment as outlined by the AAOMS.

In our case-series, we identified two patients with dental extractions concomitantly with antiresorptive treatment, and two patients with spontaneous loss of teeth secondary to severe periodontal disease. We were able to identify early-onset MRONJ and to initiate rapid treatment with broad-spectrum antibiotics and antiseptic solution underlining the importance of timely referral to an experienced odontologist.

In our institution, dental health evaluation before exposure to BMAs is performed routinely. During the dental evaluation, a meticulous assessment of oral hygiene is carried out and the patient is educated regarding the risk factors for the development of MRONJ. We emphasize on avoiding dental extractions and appeal to different approaches such as endodontics for pain-relief as delineated in the AAOMS guidelines.

Some areas of opportunity are worth noting. In Latin America, there is only one retrospective analysis by Fernández et al. on MRONJ in adult patients with cancer ^25^. In their study, 7 cases were described in 50 patients exposed to bisphosphonates in a period of 9 years; however, the study does not detail population risk factors, established therapeutic measures, description of patient outcomes, or detail adherence to AAOMS clinical treatment guidelines. Moreover, to date, there is no local adaptation of clinical guidelines for the prevention, diagnosis, and treatment of MRONJ in Latin America. Likewise, we also do not know if the general practice dentist is familiar with this complication or with the therapeutic measures recommended by the AAOMS. This lack of information is an area of opportunity to generate knowledge through clinical research. In this regard, our group is developing a cross-sectional study with the objective of making a situational diagnosis, to implement quality improvement initiatives to such as continuous dental education, highlighting the awareness of MRONJ to the general practice dentist.
